# An Office-Based Fluid to Fluid Exchange Technique for the Treatment of Postvitrectomy Vitreous Cavity Hemorrhage and Secondary Glaucoma

**DOI:** 10.1155/2017/8190823

**Published:** 2017-02-21

**Authors:** Sergio E. Hernandez-Da Mota

**Affiliations:** Clinica David, Unidad Oftalmologica, Blvd. Garcia de Leon 598-2, Colonia Nueva Chapultepec, 58280 Morelia, MICH, Mexico

## Abstract

A case of postvitrectomy hemorrhage with secondary glaucoma successfully treated with an office-based fluid to fluid exchange is described. A 25 Ga trocar was placed 3 mm from the sclerocorneal limbus at the 2 o'clock position and connected to a 250 cc elevated bottle of balanced salt solution (BSS) through an intravenous (IV) line and an infusion cannula. Afterward, a 25 Ga needle was inserted 3 mm from the limbus at the 5 o'clock position approximately. The BSS fluid entered the eye through the 25 Ga trocar lavaging the vitreous cavity and the anterior chamber. About 4 to 6 cc of hemorrhagic fluid egressed the eye through the 25 Ga needle.

## 1. Introduction

The incidence of postvitrectomy diabetic vitreous cavity hemorrhage can range from 29% to 75%, being one of the most common postoperative complications of diabetic retinopathy vitrectomy [1, 2]. Occasionally blood can enter the anterior chamber usually in pseudophakic eyes that can result in an intraocular pressure elevation.

Treatments for this condition include conservative management with antiglaucoma medications and performing another surgery to lavage the vitreous cavity [3–5].

The purpose of this case report is to describe an office-based procedure to treat recurrent postvitrectomy hemorrhage with secondary glaucoma.

## 2. Case Report

A 54-year-old female with a 15-year history of diabetes mellitus and with cataract and vitreous hemorrhage in her right eye secondary to diabetic proliferative retinopathy underwent a noncomplicated combined surgery of phacoemulsification, intraocular lens implantation, 23 Ga-three-port pars plana vitrectomy, endophotocoagulation, air-fluid exchange, and injection of SF6 gas at a nonexpansile concentration.

A month after surgery the patient had a persistent vitreous cavity hemorrhage, hyphema, and an intraocular pressure of 35 mmHg. Ultrasonography did not reveal the presence of retinal detachment.

*Surgical Procedure.* The patient received a 4 cc retrobulbar anesthetic injection of 2% lidocaine and 0.75% bupivacaine in a 50 : 50 mixture. She was positioned with her head straight in an office examination chair. After that, a sterile technique was used including a povidone-iodine solution to sterilize the conjunctival surface and eyelids, sterile gloves, mask, drape, and eyelid speculum.

**Figure 1:**
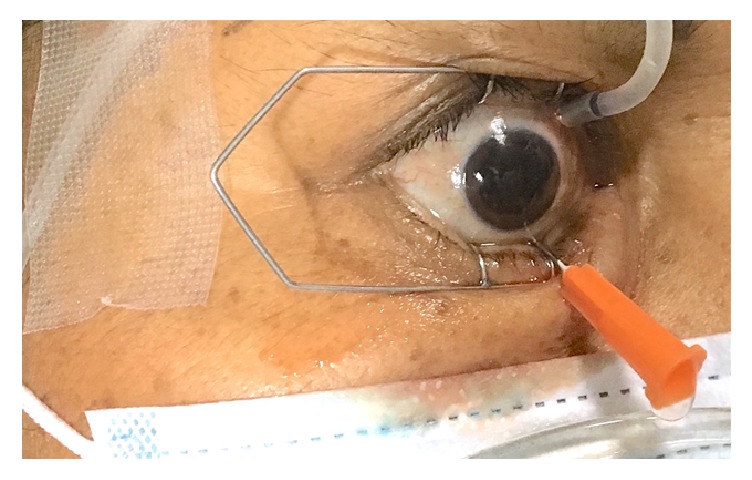
Photograph showing the infusion cannula placed superiorly 3 mm from the sclerocorneal limbus at the 2 o'clock position and the hemorrhagic fluid egressing the eye through the 25 Ga needle placed inferiorly.

**Figure 2:**
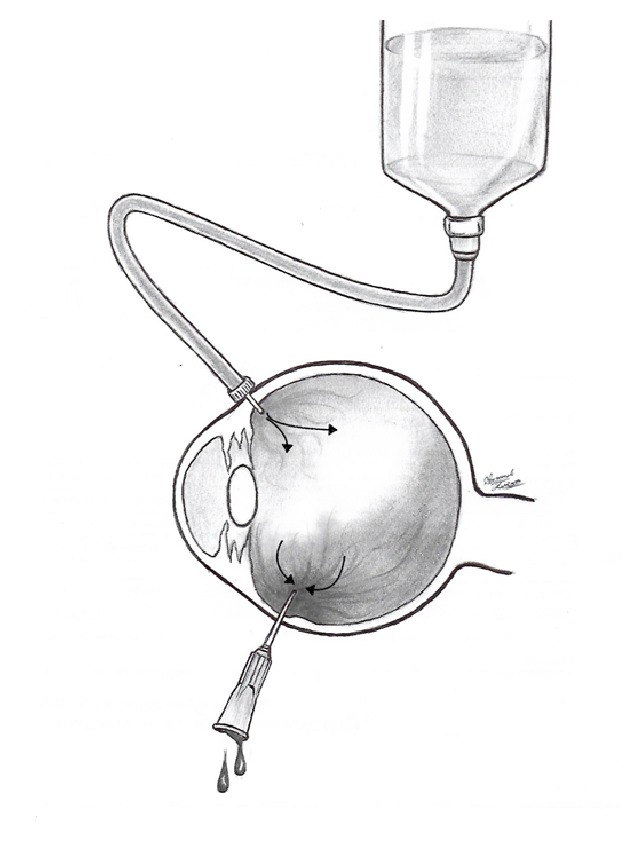
Schematic drawing showing the BSS fluid from an elevated bottle entering the eye through the infusion cannula and egressing through the needle lavaging the hemorrhagic vitreous cavity.

A 25 Ga trocar was placed 3 mm from the sclerocorneal limbus at the 2 o'clock position and connected to a 250 cc elevated bottle of balanced salt solution (BSS) through an IV line and an infusion cannula. Afterward, a 25 Ga needle was inserted 3 mm from the limbus at the 5 o'clock position approximately. The BSS fluid entered the eye through the 25 Ga trocar lavaging the vitreous cavity and the anterior chamber. About 4 to 6 cc of hemorrhagic fluid egressed the eye through the 25 Ga needle (Figures 1 and 2) and was collected in a sterile recipient. This vitreous cavity lavage was monitored with indirect ophthalmoscopy and stopped as soon as the fundus was clearly visible. At the end of the procedure, antibiotic eye drops were prescribed as infection prophylaxis.

Five days after the procedure, there was no remaining blood in the vitreous cavity nor in the anterior chamber and the intraocular pressure was 18 mmHg.

## 3. Discussion

Meticulous intraoperative control of hemorrhage and intraoperative photocoagulation are important in preventing postoperative diabetic vitreous hemorrhage. However, vitreous hemorrhage remains a common complication of diabetic vitrectomy surgery mainly in the immediate postoperative period. In the majority of cases, the magnitude of the vitreous hemorrhage is mild and does not obscure adequate fundus examination or markedly delay visual recovery [2, 3, 6].

In cases where the hemorrhage obscures the fundus and persists for more than a few weeks, other interventions might be warranted. Several techniques have been described to treat this condition. One of the most commonly performed techniques is the “push-and-pull” method [7]: a 5 to 6 ml of air or gas mixture is drawn into a 10 cc syringe, and a 27- or 30-gauge needle is utilized. The needle is inserted through the pars plana inferiorly or temporally. Once the needle tip has been directed into the midportion of the vitreous cavity, the second hand is used to direct the needle posteriorly into the globe. Fluid can be withdrawn in 0.3 to 0.5 increments and gas or air injected with one instrument, allowing intraocular fluid to drip to the bottom of the syringe, while the gas is directed from the superior portion of the syringe into the globe with gentle pressure to try to maintain normal pressure and volume relationships. This procedure is repeated in a series of steps until the entire vitreous cavity is filled with air or gas. However, the broad and sometimes painful intraocular pressure (IOP) fluctuations are a potential disadvantage of this method that the technique we describe does not have. These IOP changes can also increase the risk of recurrent and persistent vitreous cavity hemorrhages [7]. In our technique, the IOP is kept constant because the rate of fluid flow that enters the eye through the infusion cannula connected to the elevated BSS bottle is the same as the rate of fluid flow that egresses the eye. Another advantage of our technique over the “push-and-pull” method is that no gas or air is injected into the vitreous cavity that might induce the formation of small gas bubbles called “fish-eggs” and obscure the fundus.

The obscured visualization of the fundus and the formation of these small gas bubbles in the vitreous cavity often preclude the use of immediate complementary postoperative laser therapy that sometimes is necessary and may conceal complications such as new retinal breaks and subretinal gas or hemorrhage as well as the central retinal artery status [8].

With the technique we describe there is an immediate clearing of the vitreous cavity that can be monitored simultaneously either with an indirect ophthalmoscope or with a fundus camera; moreover, the patient may have a faster visual recovery.

Nonetheless, potential disadvantages of our procedure are the hazard of lens damage with the needle or the trocar in phakic eyes and a higher cost since a 25 Ga trocar and infusion cannula are used.

On the other hand, we believe that the risks of complications such as endophthalmitis are minimal since we used a sterile technique, system, and BSS to lavage the vitreous cavity.

Lastly, an effective retrobulbar anesthetic injection and case selection against anxious patients who may not tolerate the procedure in an office setting are crucial.

In conclusion, this is an efficient and quick office-based procedure for the management of postvitrectomy vitreous cavity hemorrhage with or without secondary intraocular hypertension in individual patients.
